# Surgical myomectomy followed by oral Myfembree vs standard of care (SOUL trial): Study protocol for a randomized control trial

**DOI:** 10.1371/journal.pone.0306053

**Published:** 2024-07-02

**Authors:** Samar Alkhrait, Ayman Al-Hendy, Hiba Alkelani, Theodore Karrison, Obianuju Sandra Madueke Laveaux

**Affiliations:** 1 Department of Obstetrics and Gynecology, University of Chicago, Chicago, IL, United States of America; 2 Department of Public Health Sciences, University of Chicago, Chicago, IL, United States of America; Teikyo University, School of Medicine, JAPAN

## Abstract

**Background:**

Uterine leiomyomas (often referred to as fibroids or myomas) are common benign, hormone-dependent tumors that grow in the uterus and occur in approximately 25% of reproductive age women, depending on selected population. Treatment recommendation is typically based on fibroid size, location, the patient’s age, reproductive plans, and obstetrical history. Despite the range of treatment options available for uterine fibroids and their symptoms, including hysterectomy, myomectomy, endometrial ablation, endometrial uterine artery embolization, and magnetic resonance-guided focused-ultrasound surgery, myomectomy remains the gold standard treatment for patients who desire fertility-preserving surgery for their uterine fibroids. Myomectomy, while a prevalent surgical option for the removal of fibroids, carries known risks such as fibroid recurrence, symptom recurrence, and the subsequent need for reintervention. Despite ongoing research and advances in medical treatments for fibroids, there currently are no universally recommended therapeutic interventions proven to effectively delay the recurrence of fibroids or the return of symptoms following this procedure. This situation underscores a significant area of unmet medical need and highlights the importance of continued investigation into preventive strategies and long-term management options for patients undergoing fibroid removal with uterine preservation. We designed a study to assess the efficacy of the new FDA-approved GnRH antagonist, Myfembree in delaying the return of fibroids and their associated symptoms.

**Methods:**

A randomized, prospective, open-label clinical trial. The participants (n = 136) will be randomly distributed into two groups. The Control Group (Standard of care) will receive treatment with standard of care (SoC) after surgical myomectomy and the treatment group will receive Relugolix combination therapy (Myfembree®) after surgical myomectomy. The study protocol was approved by the University of Chicago’s Institutional Review Board (IRB#22–0282), ensuring that all participants would provide written informed consent before their inclusion.

**Discussion:**

In this project, we propose the use of daily dosed Relugolix combination therapy (Relugolix with estradiol and norethindrone acetate), which is approved for uterine fibroids treatment, has the potential to delay the recurrence of fibroid symptoms, prolong the improved quality of life and delay need for re-intervention after uterine sparing surgery.

**Trial registration:**

The study protocol was approved by the Institutional Review Board of the University of Chicago on 9/16/2022 and was registered at ClinicalTrials.gov with number NCT05538689 on Sep 7, 2022. All subjects will provide informed consent to participate.

## Introduction

Uterine leiomyomas (often referred to as fibroids or myomas) are common benign, hormone-dependent tumors that grow in the uterus and occur in approximately 25% of women of reproductive age, depending on selected population. While most uterine fibroids are asymptomatic, approximately 25% of women with fibroids develop symptoms requiring treatment [1]. The most problematic symptom for women with uterine fibroids is abnormal uterine bleeding (AUB), with menstrual periods of increased duration and volume [2].

Persistent AUB can induce iron-deficiency anemia and associated fatigue and loss of energy. AUB is a primary reason for the deterioration in the health-related quality of life assessed in patients with uterine fibroids and is a major cause of elective hysterectomy. Other symptoms include bulk symptoms, such as pain or pressure in the abdomen and pelvis due to large myoma(s), low back pain, urinary frequency or urinary tract obstruction, constipation, and adverse perinatal outcomes [3].

The type of treatment recommended for uterine fibroids typically depends upon their size, location, the patient’s age, reproductive plans, and obstetrical history. The decided approach points to the important role of individually tailored therapy. Until recently, only a few medical options were available for women with AUB. Most of the medical therapies produce temporary reduction in both uterine size and fibroid symptoms. A 2016 Cochrane systematic review on surgery versus medical therapy for heavy menstrual bleeding (HMB) showed that 59% of women randomized to the oral medication group had had surgery within two years and 77% within five years [4].

As a direct result of this, the current mainstay of treatment for women with AUB is surgery. Several surgical procedures are frequently performed including hysterectomy, myomectomy by various techniques, endometrial ablation, endometrial uterine artery embolization, and magnetic resonance-guided focused-ultrasound surgery. Each of these options has risks and benefits which must be carefully considered by both provider and patient in a shared decision-making process.

In 2020, a new drug–Oriahnn™, a non-peptide gonadotropin-releasing hormone (GnRH) hormone antagonist ‐ Elagolix combined with estradiol and norethindrone acetate, received US Food and Drug Administration (FDA) approval for the management of AUB-L for up to 24 months. Even more recently, in 2021, relugolix combination therapy (relugolix with estradiol and norethindrone acetate, Myfembree®) received FDA approval for AUB-L in premenopausal women, for up to two years [3]. According to available data Myfembree®, outperformed its placebo comparison groups with a total of 73% and 71% of study participants receiving relugolix combination therapy as compared to 19% and 15% of those in placebo groups, respectively, achieving the primary efficacy endpoint ‐ volume of menstrual blood loss <80 ml and a ≥50% reduction in volume from baseline. Patients receiving Myfembree® versus placebo also showed significant improvements in six of seven key secondary endpoints ‐ measures of menstrual blood loss (including amenorrhea), pain, distress from bleeding and pelvic discomfort, anemia, and uterine volume, but not fibroid volume. These long-term, well-tolerated medical therapies are the first group of medications designed specifically for AUB-L and represent a changing paradigm in fibroid treatment. Of critical note is that unlike with Elagolix combination therapy, Oriahnn™, the use of Relugolix combination therapy, Myfembree® significantly reduced fibroid-related pain measured with the use of a daily electronic diary and a validated pain-outcome measure [3].

Management of symptomatic uterine fibroids often requires a combination of treatment options to achieve desired outcomes. These options may include medical–hormonal, antifibrinolytics, non-steroid anti-inflammatory drugs (NSAIDs); procedural–uterine fibroid embolization, high-intensity focused ultrasound (HIFU) ablation, radiofrequency ablation (RFA) and surgical–myomectomy or hysterectomy. For patients who opt for surgical treatment, they must choose between uterine preservation and definitive surgery i.e., hysterectomy. Myomectomy is the only uterine-sparing surgical treatment of fibroid in which the fibroid is surgically excised from the uterus. Myomectomy is the recommended option for women with large and bulky fibroids who want to retain the option for future fertility or have strong personal or cultural reasons to retain their uterus. It is important to understand that the goal of a myomectomy is not to excise all fibroids but to strategically excise the fibroids that are clinically relevant based on size and/or location. As a result, women who undergo myomectomy, have a known risk of symptom recurrence and subsequent need for reintervention [5].

There are two main proposed theories to explain fibroid recurrence after myomectomy. 1. The growth of small residual fibroids that were strategically retained within the uterus at the time of initial myomectomy [6]. 2. The natural evolution of fibroid-adjacent myometrium leading to initiation and continued proliferation of fibroids [6]. A retrospective cohort study of over 35,000 women undergoing myomectomy, endometrial ablation, and uterine artery ablation showed a 24-month reintervention rate of 4.2% and a 5-year reintervention rate of 19% (17%, 28%, and 20% for abdominal, hysteroscopic, laparoscopic approach, respectively) [7]. In 2001, Rossetti et al., in a study comparing the risk of symptom recurrence after laparoscopic versus abdominal myomectomy, reported a crude rate of recurrence of 27%, and most recurrences were detected by sonography between 10 and 30 months postoperatively [8]. Meanwhile, Nezhat et al. reported a cumulative risk of recurrence of 10.6% after 1 year, 31.7% after 3 years, and 51.4% after 5 years [9]. In a 2020 study comparing the long-term symptom alleviation and re-intervention after HIFU ablation and secondary myomectomy for women with recurrent symptomatic uterine fibroids following myomectomy, the cumulative risk for re-intervention after secondary myomectomy was 3.2% at 1 year and 11.9% at 3 years [10]. Clinical factors that reportedly impact the risk of reoperation after laparoscopic myomectomy due to leiomyoma recurrence include age, number and size of fibroids, size of uterus, type of surgery, parity after myomectomy and use of medical therapies such as GnRH analogs [11]. Fibroid recurrence after laparoscopic myomectomy may be assessed by a) evaluating clinical signs or symptoms, b) screening ultrasound investigation, c) self-reported symptoms based on questionnaires d) focused ultrasound investigation based on clinical exam [12].

Despite the risk of recurrent symptoms and the need for reintervention which is reported by some authors to be as high as 60% after 5 years [13], there have been no clinical trials investigating agents to minimize this risk. As a result, there are no clear recommendations on methods to delay the recurrence of symptoms, growth of additional fibroids, and ensure a sustained improvement in quality of life.

## Methods

### Study design and location

This will be a randomized, single-center, open-label interventional clinical trial of treatment with Relugolix combination therapy (Myfembree®) after surgical myomectomy vs. Treatment with standard of care after surgical myomectomy. The standard of care will depend on the type of surgery, health history, and clinical symptoms. It often includes pain management, bleeding management, physical exams, and pelvic ultrasound. The recruitment will be from the University of Chicago Medical Center in Illinois USA. The randomization scheme will be coordinated by the Biostatistics Laboratory (located within the Department of Public Health Sciences) at the University of Chicago. The study protocol was approved by the Institutional Review Board of the University of Chicago on 9/16/2022 (IRB22-0282) and the initial recruitment of participants commenced on February 2, 2023, with a written consent form being obtained from each participant. The study is anticipated to conclude in February 2026.

**Fig 1:**
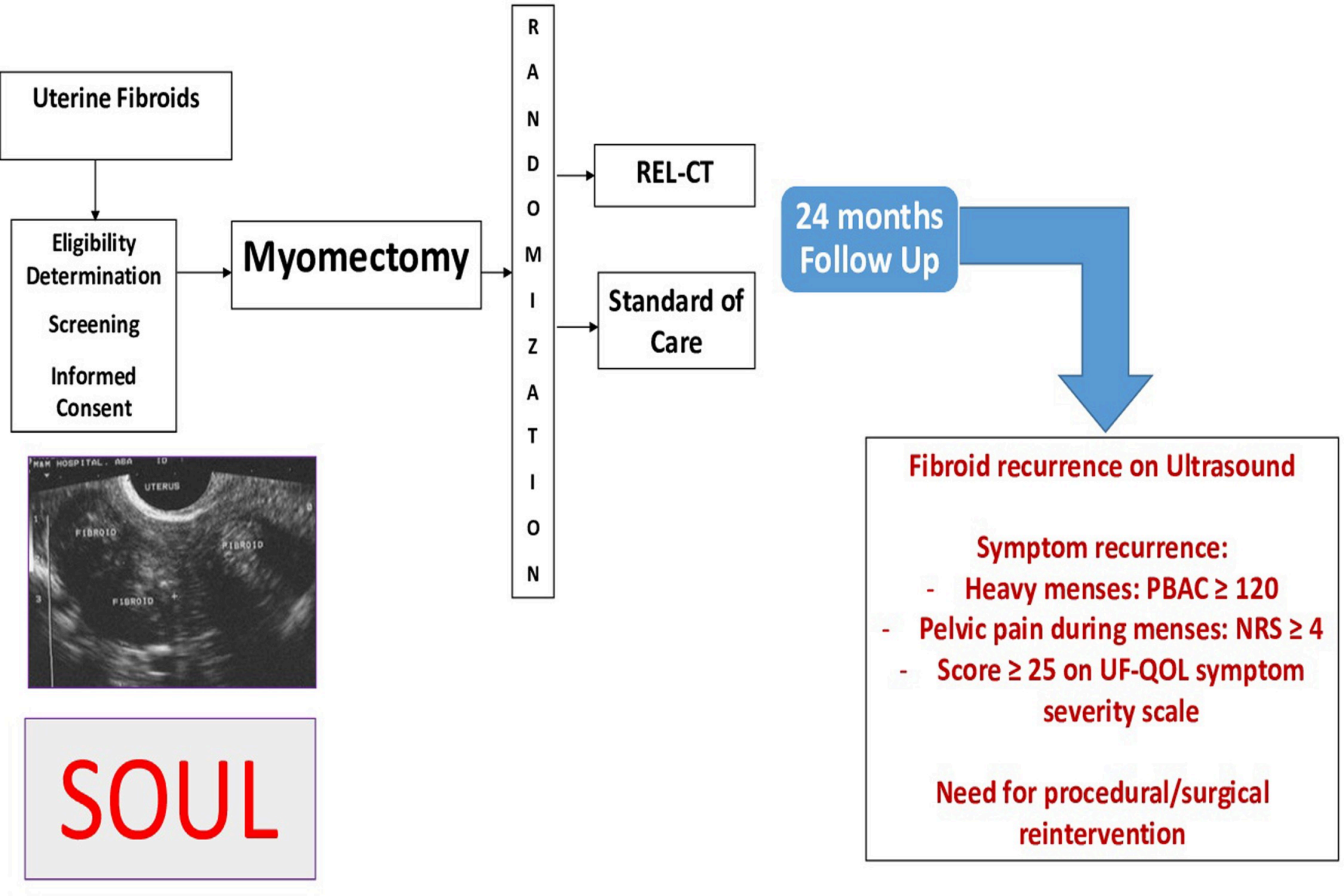
Overall study design represents the study’s flow from enrollment through various interventions to the endpoints over 24 months.

Fig 1 summarizes the study design.

### Recruitment

A member of the research team will search the University of Chicago operating room schedules of the surgeons to identify potential study participants scheduled to undergo a myomectomy. Data from the medical record system will be used to evaluate inclusion criteria. Patients presenting to the clinic for a consultation for fibroid symptoms or fibroid management (after clinical confirmation of their eligibility for admission into the study based on inclusion & exclusion criteria) will also be considered.

Once a potential participant is identified, they will be approached to participate by a member of the research team. Contact is established via MyChart, email, phone number, in person at the clinic, or pre-op. Eligibility criteria will be confirmed by reviewing medical history and thorough discussion with potential subjects. If eligibility is confirmed, the potential subject will be offered participation in the study. At this time, study details will be explained. Risks and benefits will be thoroughly discussed, and consent given to the participant.

### Inclusion criteria

1. Has voluntarily signed and dated the informed consent form prior to initiation of any screening or study-specific procedures
2. Premenopausal female aged 18 years and older on the day of signing of the informed consent form
3. Has a diagnosis of uterine fibroids that is confirmed by a pelvic ultrasound (transvaginal and/or transabdominal) performed during the screening period.
4. Has at least one or more of the following symptoms:
  - Moderate to heavy menses defined as PBAC score ≥ 120.
  - Pelvic pain during menses measured on NRS ≥ 4 at baseline.
  - Moderately severe fibroid-related symptoms (a score ≥ 25 on the UF quality of life symptoms severity subscale)
5. Has a negative urine pregnancy test at the Screening, Baseline and interval clinic visits
6. Agrees to not be pregnant for at least 12 months. Participants may use any form of non-hormonal contraception consistently during the screening period and the randomized treatment period. These may include Diaphragm, cervical cap, spermicides, male and female condoms, copper IUD and sponge.
7. Has an endometrial (aspiration) biopsy, if clinically indicated, performed during the screening period, with results showing no clinically significant endometrial pathology (hyperplasia, endometritis, or endometrial cancer).

### Exclusion criteria

1. Has transvaginal and/or transabdominal ultrasound during the screening period demonstrating pathology other than uterine fibroids that could be responsible for or contributing to the patient’s heavy menstrual bleeding, such as uterine or cervical polyps > 2.0 cm, or any other clinically significant gynecological disorder determined by the investigator to require further evaluation and/or treatment.
2. Has unexplained vaginal bleeding outside of the patient’s regular menstrual cycle
3. Has undergone ultrasound-guided laparoscopic radiofrequency ablation, or any other surgical procedure for fibroids, uterine artery embolization, magnetic resonance-guided focused ultrasound for fibroids, as well as endometrial ablation for abnormal uterine bleeding within 6 months prior to the Screening visit
4. Has a history of or currently has osteoporosis, or other metabolic bone disease, hyperparathyroidism, hyperprolactinemia, hyperthyroidism, anorexia nervosa, or low traumatic (from the standing position) or atraumatic fracture (toe, finger, skull, face, and ankle fractures are allowed). A history of successfully treated hyperparathyroidism, hyperprolactinemia, or hyperthyroidism is allowed if the patient’s bone mineral density is within normal limits.
5. Has a history of the use of bisphosphonates, calcitonin, calcitriol, ipriflavone, teriparatide, denosumab, or any medication other than calcium and vitamin D preparations to treat bone mineral density loss
6. Anticipated use of systemic glucocorticoids at an oral prednisone-equivalent dose of more than 5 mg every other day during the study. Note: topical, inhaled, intranasal, optic, ophthalmic, intraarticular, or intralesional subcutaneous are permitted without restriction
7. Gastrointestinal disorder affecting absorption or gastrointestinal motility
8. Has any additional contraindication to treatment with low-dose estradiol and norethindrone acetate, including:
  - Current, known, suspected, or history of breast cancer.
  - Current, known, or suspected hormone-dependent neoplasia
  - High risk of arterial, venous thrombotic disorder or thromboembolic disorder, i.e. women over 35 years of age who smoke or women with uncontrolled hypertension.
  - Known anaphylactic reaction or angioedema or hypersensitivity to estradiol or norethindrone acetate.
  - Currently pregnant or lactating or intends to become pregnant or to donate ova during the study period or within 1 month after the end of the study.
  - Active thrombotic or thromboembolic disease or history of these conditions prior to the Baseline Day 1 visit or risk factors for such conditions. These conditions include:
    - deep vein thrombosis
    - pulmonary embolism
    - vascular disease (e.g., cerebrovascular disease, coronary artery disease, peripheral vascular disease)
    - inherited or acquired hypercoagulopathies, known protein C, protein S, or antithrombin deficiency, or other known thrombophilia disorders, including Factor V Leiden thrombogenic valvular or thrombogenic rhythm diseases of the heart (for example, subacute bacterial endocarditis with valvular disease, or atrial fibrillation)
    - uncontrolled hypertension
    - headaches with focal neurological symptoms or migraine headaches with aura if over 35 years of age
    - Women at increased risk for thrombotic or thromboembolic events
9. Has jaundice or known current active liver disease from any cause, including hepatitis A (HAV IgM), hepatitis B (HBsAg), or hepatitis C (HCV Ab positive, confirmed by HCV RNA)
10. Has any of the following cervical pathology: high-grade cervical neoplasia, atypical glandular cells, atypical endocervical cells, atypical squamous cells favoring high grade. Of note, patients with atypical squamous cells of undetermined significance and low-grade cervical neoplasia may be included in the study if high-risk human papillomavirus testing is negative or if DNA testing for human papillomavirus 16 and 18 DNA testing is negative.
11. Has any history of clinical laboratory abnormalities indicating hepatic or gallbladder impairment.
12. Has clinically significant cardiovascular disease including:
  - Prior history of myocardial infarction
  - History of angina
  - History of congestive heart failure
  - History of clinically significant ventricular arrhythmias such as ventricular tachycardia, ventricular fibrillation, or torsade de pointes, or Mobitz II second-degree or third-degree heart block without a permanent pacemaker in place or untreated supraventricular tachycardia (heart rate ≥ 120 beats per minute)
  - QT interval by the Fridericia correction formula (QTcF) of > 470 msec
  - Hypotension, as indicated by systolic blood pressure < 84 millimeters of mercury (mmHg) on 2 repeat measures at least 15 minutes apart or treated ongoing symptomatic orthostatic hypotension with > 20 mmHg decrease in systolic blood pressure one minute or more after assuming an upright position.
  - Uncontrolled hypertension, as indicated by systolic blood pressure > 160 mmHg on 2 repeat measures at least 15 minutes apart or diastolic blood pressure > 100 mmHg at any screening visit or the Baseline Day 1 visit.
  - Bradycardia as indicated by a heart rate of < 45 beats per minute on the screening electrocardiogram.
13. Has been a participant in an investigational drug or device study within the 1 month prior to the screening visit.
14. Has a history of clinically significant condition(s) including, but not limited to:
  - Untreated thyroid dysfunction or palpable thyroid abnormality (patients with adequately treated hypothyroidism who are stable on medication are not excluded).
  - History of malignancy within the past 5 years or ongoing malignancy other than curatively treated nonmelanoma skin cancer or surgically cured Stage 0 in situ melanoma
15. Any current psychiatric disorder that would, in the opinion of the investigator or medical monitor, impair the ability of the patient to participate in the study or would impair the interpretation of their data.
16. Has a prior (within 1 year of Screening 1 visit) or current history of drug or alcohol abuse disorder according to Diagnostic and Statistical Manual of Mental Disorders V (all patients must be questioned about their drug and alcohol use, and this should be documented in the electronic case report form)
17. Has participated in a previous clinical study that included the use of Relugolix or has received this treatment within 3 months of the study.
18. Is inappropriate for participation in this study for other reasons, as determined by the investigator, sub-investigator, or medical monitor.

### Randomization

Randomization will be prepared by the study statistician using computer-generated random numbers and the method of permuted blocks. It will be implemented using the REDCap randomization module with varying blocks of size 4, 6, and 8 (in a random order) [14]. No one other than the statistician will have access to the sequence of block sizes. Eligible patients will be assigned to one of the following arms:

Arm 1: Treatment with Relugolix combination therapy (Myfembree®) after surgical myomectomy.
Arm 2: Treatment with standard of care (SoC) after surgical myomectomy.

### Variables

The primary outcome will be a composite outcome including:

1. Fibroid recurrence when compared to the post-myomectomy baseline pelvic ultrasound (transvaginal and/or transabdominal), defined as a new fibroid identified on ultrasound with volume >1 cm3 [8]
2. Symptom recurrence defined as either of the following:
  I. Moderate to heavy menses with PBAC score ≥ 120 for patients with pre-operative heavy menses
  II. Pelvic pain during menses as measured on the Numerical Rating Scale NRS ≥ 4 at baseline
  III. Score ≥ 25 on the symptom severity scale.
3. Need for reintervention defined as need to undergo a procedure or surgery for treatment of uterine fibroids including repeat myomectomy, uterine fibroid embolization, radiofrequency ablation or hysterectomy.

The secondary outcomes will include:

1. Fibroid recurrence, compared to baseline pelvic ultrasound (transvaginal and/or transabdominal), defined as a new fibroid identified on ultrasound with volume >1 cm3.
2. Return of moderate to heavy menses defined as PBAC score ≥ 120 for patients with pre-operative moderate to heavy menses.
3. Need for reintervention defined as need to undergo a procedure or surgery for treatment of uterine fibroids including repeat myomectomy, uterine fibroid embolization, and radiofrequency ablation.
4. Time to event (procedure or surgery) such as radiofrequency ablation (RFA), myomectomy, hysterectomy, or uterine fibroid embolization (UFE).
5. Symptom recurrence according to the UF quality of life symptoms severity subscale and Bleeding & pelvic discomfort (BPD) scale.
6. Return of pelvic pain during menses as measured on NRS [15]
7. Quality of life as measured by Patient Reported Outcome and Quality of life questionnaires (UFS-QOL, WPAI:SHP, Female Sexual Function Index)

### Intervention

At 4–6 weeks post-surgery, participants will complete a baseline ultrasound. Participants in the medication arm will receive a once-daily pill that contains Relugolix (a gonadotropin-releasing hormone antagonist), estradiol (an estrogen), and norethindrone acetate (a progestin). Myfembree will be dispensed after randomization at study visit 1. Pill counts and checks will be completed at each visit. A total of 10 visits are necessary including the screening visit.

All participants will be asked to fill out questionnaires at baseline and then every 3 months. The questionnaires will be filled by paper or electronically using uMotif e-diary.

- Uterine Fibroid Symptom and Health-Related Quality of Life (UFS-QoL) fibroid Questionnaire
- Work Productivity and Activity Impairment Questionnaire: Specific Health Problem V2.0 (WPAI:SHP)
- Female Sexual Function Index (FSFI)

All participants will be asked to fill a set of diaries at baseline and daily for the study duration.

- Daily electronic diaries (eDiary) to measure compliance with study treatment
- Menstrual bleeding diary
- Record of use of feminine products for menstrual bleeding
- Pictorial bleeding assessment chart (PBAC) scores
- Pelvic pain during menses by the numeric rating scale (NRS)
- Use of pain medication to treat pain caused by uterine fibroids

Participants will have access to all questionnaires once they are enrolled in the study and they will be accessible anywhere through their phones, mobile, or computer devices.

All participants will undergo pelvic ultrasounds at the following time periods:

- Week 4–6: post-myomectomy baseline ultrasound (transvaginal and/or transabdominal)
- Month 6: pelvic ultrasound (transvaginal and/or transabdominal)
- Month 12: pelvic ultrasound (transvaginal and/or transabdominal)
- Month 18: pelvic ultrasound (transvaginal and/or transabdominal)
- Month 24: pelvic ultrasound (transvaginal and/or transabdominal)

All participants will have blood work performed every 6 months or at clinically indicated intervals:

- Cell Blood Count (CBC)
- Complete Metabolic Panel (CMP)
- Urine pregnancy test during each clinic/in-person visit.

**Fig 2:**
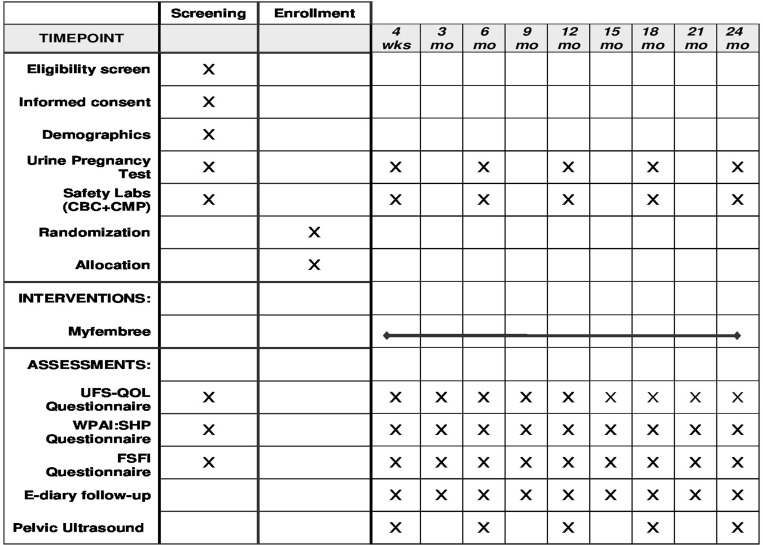
Timeline and Interventions Illustrate the structured timeline and intervention schematic for a 24-month clinical study, detailing enrollment, interventions, and assessments including UFS-QOL and pelvic ultrasounds at specified time points.

Please refer to Fig 2 for a detailed study timeline & interventions.

### Sample size and statistical analysis

Data analysis and statistical support is obtained through the Biostatistics Laboratory (located within the Department of Public Health Sciences) at the University of Chicago. The primary endpoint of this study will be a composite endpoint defined as the occurrence of any of the following three component endpoints over a 24 month follow up period.

Endpoint 1 (Fibroid Recurrence)

Endpoint 2 (Symptom Recurrence)

Endpoint 3 (Need for Re-intervention)

A Chi-square test will be used to compare the rate of occurrence of the primary endpoint in the two treatment arms.

Assumptions regarding the frequency of endpoint 1 are based on ref [8]. That study observed 23% recurrences in the abdominal group and 27% in the laparoscopic group over 40 months. 84% of these recurrences were seen within 24 months. Therefore, we assume the recurrence rate among patients with laparoscopic or abdominal surgery over 24-month follow-up time in the control arm will be (23%+27%)/2*0.84 = 21%. We assume a recurrence rate of 5% in the treatment group (i.e., effect size of 16% absolute reduction). For endpoint 2, a symptom recurrence rate of 7.6% in the control group [16], and 1.5% in the treatment group are assumed. For endpoint 3, a reintervention rate of 14.4% [16] in the control group and 3% in the treatment group are assumed. Assuming endpoint 1 is independent of endpoints 2 and 3, and a correlation of 0.5 between the endpoints 2 and 3, leads to a rate of 33.8% for our primary composite endpoint in the control arm vs. 8.25% in the treatment group. A sample size of 94 patients (47 per arm) will provide 80% power to detect a difference of this magnitude at a two-sided alpha level of 0.05 (PASS, Kaysville, Utah) utilizing the chi-square test as the statistical testing method. To allow for a 30% dropout rate, the sample size will be increased to 136 patients. It is expected that the 136 patients will be accrued during the first year of the trial (10–12 per month) with two years of follow-up after the last patient is enrolled. The number needed to treat (NNT) will be calculated as the reciprocal of the difference in observed event rates along with a 95% confidence interval. While the sample size has been increased to allow for a 30% dropout rate, every effort will be made to retain subjects in the trial and to obtain outcome data. Sensitivity analyses will be performed to examine the effects of dropouts on the results.

As secondary endpoints, we will analyze the three components of the primary endpoint (recurrence, symptoms, and re-intervention) separately using Chi-square or Fisher’s exact tests. Adverse event (AE) rates will be summarized by type, grade, and attribution and monitored periodically by the investigative team and compared between groups using Chi-square or Fisher’s exact tests. All categorical data will be summarized as frequencies and percentages. Pain scores and quality of life (QOL) measures over time will be summarized as means and standard deviations or medians and interquartile ranges depending on the shape of the distribution. Comparisons between groups will be performed using mixed effects models for longitudinal data [14]. Empirical semi-variograms will be constructed to assess the underlying covariance structure together with information criteria (AIC). If a suitable parametric form (for example, AR(1)) cannot be identified we will utilize an unstructured variance-covariance structure. Finally, Kaplan-Meier curves will be generated for time-to-event outcomes; logrank tests will be performed for group comparisons. REDCap will be used for data capture and data management while SAS (Raleigh, NC) and STATA (College Station, TX) will be the principal software employed for data analysis.

### Confidentiality of study data

To ensure the confidentiality of medical information, each patient will be assigned a unique identifier in the database that can be linked to the medical record number. The database will be password-protected, encrypted, and stored on a secure server accessible only from computers in the OBGYN department. Subject demographics and dates will be entered into REDCap (system 4283). REDCap is a mature and secure web application for building and managing online surveys and databases. It allows data to be exported to Excel or R or SPSS. REDCap has the capacity to allow patients to securely link to consent forms. The electronic consent form will be accessed via an email link. REDCap is HIPAA compliant, and data is encrypted during transmission, allowing for data collection out-of-network, off-campus, remotely, etc. Patients are able to sign the consent form with the use of a mouse or a touch screen.

UMotif is BSI-certified and operates a quality management system that is ISO9001-2015 compliant. It is used for patient-centric software as a service platform to capture electronic clinical outcome assessment data in a healthcare setting including the healthcare industry.

## Supporting information

S1 Checklist SPIRIT 2013 checklist: Recommended items to address in a clinical trial protocol and related documents*. (DOC)

S1 Protocol (PDF)
