# Supplementary material for: Surgical myomectomy followed by oral Myfembree vs standard of care (SOUL trial): Study protocol for a randomized control trial
Source: PLoS One. 2024 Jul 2;19(7):e0306053. doi: 10.1371/journal.pone.0306053 (PMC11218990; doi:10.1371/journal.pone.0306053)
Supplement: S1 Protocol — (PDF) [file pone.0306053.s002.pdf]

Research Study Protocol

Title: Surgical Myomectomy Followed by Oral Myfembree Versus Standard of Care Trial (SOUL Trial)

Abbreviated Title: SOUL Study

Principal Investigator(s):
Obianuju Sandra Madueke-Laveaux, MD MPH
Assistant Professor, Department of Obstetrics & Gynecology
Section of Minimally Invasive Gynecologic Surgery
[redacted]

Table of Contents

ACRONYMS **3**

INTRODUCTION **4**

1.0 STUDY PURPOSE AND RATIONALE **5**

2.0 STUDY DESIGN AND STATISTICAL PROCEDURES **6**

2.1 Overall design 6
2.2 Study objectives 6
2.3 Aims & Hypotheses 7
2.4 Data Management and Statistical Analysis 8
2.5 Graphical schema of study 9

3.0 STUDY PROCEDURES **10**

4.0 STUDY DRUGS **12**

5.0 STUDY INSTRUMENTS: Questionnaires (PDF attached to IRB application portal) **12**

6.0 STUDY SUBJECTS **12**

6.1 Inclusion criteria: 12
6.2 Exclusion criteria: 13

7.0 RECRUITMENT **15**

8.0 INFORMED CONSENT PROCESS **16**

9.0 CONFIDENTIALITY OF STUDY DATA **18**

10.0 PRIVACY PROTECTIONS **18**

11.0 POTENTIAL RISKS **18**

12.0 ADVERSE EVENTS DEFINITION **21**

13.0 DATA AND SAFETY MONITORING **23**

14.0 POTENTIAL BENEFITS **24**

15.0 ALTERNATIVES **24**

16.0 RESEARCH AT EXTERNAL SITES **24**

17.0 UNIVERSITY OF CHICAGO AS LEAD INSTITUTION **24**

Proposed Timeline **24**

Bibliography ***25***

ACRONYMS

SOUL Trial: Surgical Myomectomy Followed by Oral Myfembree Versus Standard of Care Trial
UF: Uterine Fibroids
AUB-L: Abnormal Uterine Bleeding
HMB: heavy menstrual bleeding
FDA: Food and Drug Administration
GnRH analogues: Gonadotropin-releasing hormone analogues
NSAIDs: non-steroid anti-inflammatory drugs
HIFU ablation: high-intensity focused ultrasound ablation
RFA: radiofrequency ablation
UFE: uterine fibroid embolization
BPD scale: Bleeding & pelvic discomfort scale
NRS: Numerical Rating Scale (pain scale)
PBAC Score: Pictoral Blood Loss Assessment Chart
QOL: Quality of Life
UFS-QOL: Uterine Fibroid Symptom & Health-Related Quality Of Life Questionnaire
WPAI:SHP: Work Productivity and Activity Impairment Questionnaire
FSFI: Female Sexual Function Index
eDiary: Electronic Diaries
CBC: Cell Blood Count
CMP: Complete Metabolic Panel
REDCap: Research Electronic Data Capture
HIPAA: Health Insurance Portability and Accountability Act
IRB: Institutional Review Board
PHI: Protected Health Information
PI: Principal Investigator
SOC: Standard of Care
U of C: University of Chicago
SAE: Serious Adverse Event
MedDRA: Medical Dictionary for regulatory activities
DCAM: UChicago Medicine Duchoossois Center for Advanced Medicine

INTRODUCTION

Uterine leiomyomas (often referred to as fibroids or myomas) are common benign, hormone-dependent tumors that grow in the uterus and occur in approximately 25% of women of reproductive age, depending on selected population. While most uterine fibroids are asymptomatic, approximately 25% of women with fibroids develop symptoms requiring treatment (Stewart, 2001). The most problematic symptom for women with uterine fibroids is abnormal uterine bleeding (AUB-L), with menstrual periods of increased duration and volume. (Munro MG, 2010)

Persistent AUB-L can induce iron-deficiency anemia and associated fatigue and loss of energy. AUB is a primary reason for the deterioration in the health-related quality of life assessed in patients with uterine fibroids and is a major cause of elective hysterectomy. Other symptoms include bulk symptoms, such as pain or pressure in the abdomen and pelvis due to large myoma(s), low back pain, urinary frequency or urinary tract obstruction, constipation, and adverse perinatal outcomes (Al-Hendy, Lukes, Poindexter, Venturella, & Villarroel, 2021).

The type of treatment recommended for uterine fibroids typically depends upon their size, location, the patient's age, reproductive plans, and obstetrical history. The decided approach points to the important role of individually tailored therapy. Until recently, only a few medical options were available for women with AUB-L. Most of the medical therapies produce temporary reduction in both uterine size and fibroid symptoms. A 2016 Cochrane systematic review on surgery versus medical therapy for heavy menstrual bleeding (HMB) showed that 59% of women randomized to the oral medication group had had surgery within two years and 77% within five years (Marjoribanks, Lethaby, & Farquhar, 2016).

As a direct result of this, the current mainstay of treatment for women with AUB-L is surgery. Several surgical procedures are frequently performed including hysterectomy, myomectomy by various techniques, endometrial ablation, endometrial uterine artery embolization, and magnetic resonance-guided focused-ultrasound surgery. Each of these options has risks and benefits which must be carefully considered by both provider and patient in a shared decision-making process.

In 2020, a new drug – Oriahnn™, a non-peptide gonadotropin-releasing hormone (GnRH) hormone antagonist - Elagolix combined with estradiol and norethindrone acetate, received US Food and Drug Administration (FDA) approval for the management of AUB-L for up to 24 months. Even more recently, in 2021, relugolix combination therapy (relugolix with estradiol and norethindrone acetate, Myfembree®) received FDA approval for AUB-L in premenopausal women, for up to two years (Al-Hendy, Lukes, Poindexter, Venturella, & Villarroel, 2021). According to available data Myfembree®, outperformed its placebo comparison groups with a total of 73% and 71% of study participants receiving relugolix combination therapy as compared to 19% and 15% of those in placebo groups, respectively, achieving the primary efficacy endpoint - volume of menstrual blood loss <80 ml and a ≥50% reduction in volume from baseline. Patients receiving Myfembree® versus placebo also showed significant improvements in six of seven key secondary endpoints - measures of menstrual blood loss (including amenorrhea), pain, distress from bleeding and pelvic discomfort, anemia, and uterine volume, but not fibroid volume. These long-term, well- tolerated medical therapies are the first group of medications designed specifically for AUB-L and represent a changing paradigm in the fibroid treatment. Of critical note is that unlike with Elagolix combination therapy, Oriahnn™, use of Relugolix combination therapy, Myfembree® significantly reduced fibroid-related pain measured with the use of a daily electronic diary and a validated pain-outcome measure (Al-Hendy, Lukes, Poindexter, Venturella, &

Villarroel, 2021).

1.0 STUDY PURPOSE AND RATIONALE

Management of symptomatic uterine fibroids often requires a combination of treatment options to achieve desired outcomes. These options may include medical – hormonal, antifibrinolytics, non-steroid anti-inflammatory drugs (NSAIDS); procedural – uterine fibroid embolization, high-intensity focused ultrasound (HIFU) ablation, radiofrequency ablation (RFA) and surgical – myomectomy or hysterectomy. For patients who opt for surgical treatment, they must make a choice between uterine preservation and definitive surgery i.e., hysterectomy. Myomectomy is the only uterine sparing surgical treatment of fibroid in which the fibroid is surgically excised from the uterus. Myomectomy is the recommended option for women with large and bulky fibroids who want to retain the option for future fertility or have strong personal or cultural reasons to retain their uterus. It is important to understand that the goal of a myomectomy is not to excise all fibroids but to strategically excise the fibroids that are clinically relevant based on size and/or location. As a result, women who undergo myomectomy, have a known risk of symptom recurrence and subsequent need for reintervention (Sukur, Kankaya, & Ates, 2015).

There are two main proposed theories to explain the fibroid recurrence after myomectomy. 1. The growth of small residual fibroids that were strategically retained within the uterus at the time of initial myomectomy (Fedele, Vercellini, & Bianchi, 1990). 2. The natural evolution of fibroid-adjacent myometrium leading to initiation and continued proliferation of fibroids (Candiani , Fedele, & Parazzini, 1991). A retrospective cohort study of over 35,000 women undergoing myomectomy, endometrial ablation and uterine artery ablation showed a 24-month reintervention rate of 4.2% and a 5-year reintervention rate of 19% (17 %, 28% and 20% for abdominal, hysteroscopic, laparoscopic approach, respectively) (Davis, Soliman, & Castelli-Haley, 2018). In 2001, Rossetti et al., in a study comparing risk of symptom recurrence after laparoscopic versus abdominal myomectomy, reported a crude rate of recurrence of 27%, and most recurrences were detected by sonography between 10 and 30 months postoperatively (Rossetti, Sizzi, & Soranna, 2001). Meanwhile, Nezhat et al. reported a cumulative risk of recurrence of 10.6% after 1 year, 31.7% after 3 years, and 51.4% after 5 years (Nezhat, Roemisch, & Nezhat, 1998). In a 2020 study comparing the long-term symptom alleviation and re-intervention after HIFU ablation and secondary myomectomy for women with recurrent symptomatic uterine fibroids following myomectomy, the cumulative risk for re-intervention after secondary myomectomy was 3.2% at 1 year and 11.9% at 3 years. (Liu, Tang, & Luo, 2020)

Clinical factors that reportedly impact the risk of reoperation after laparoscopic myomectomy due to leiomyoma recurrence include age, number and size of fibroids, size of uterus, type of surgery, parity after myomectomy and use of medical therapies such as GnRH analogues (Fauconnier, Chapron, & Babaki-Fard, 2000). Fibroid recurrence after laparoscopic myomectomy may be assessed by a) evaluating clinical signs or symptoms, b) screening ultrasound investigation, c) self-reported symptoms based on questionnaires d) focused ultrasound investigation based on clinical exam (Lee & Wang, 2009).

Despite the risk of recurrent symptoms and need for reintervention which is reported by some authors to be as high as 60% after 5 years, (Donnez, Donnez, & Dolmans, 2014) there have been no clinical trials investigating agents to minimize this risk. As a result, there are no clear recommendations on methods to delay recurrence of symptoms, growth of additional fibroids and ensure a sustained improvement in quality of life. In this project we **propose** that use of daily dosed Relugolix combination therapy (Relugolix with estradiol and norethindrone acetate) which

is approved for uterine fibroids treatment, has the potential to delay recurrence of fibroid symptoms, prolong improved quality of life and delay need for re-intervention after uterine sparing surgery.

2.0 STUDY DESIGN AND STATISTICAL PROCEDURES

2.1 Overall design

Type of study: A randomized open label-controlled trial

Randomization: Randomization will be prepared by the study statistician using computer-generated random numbers and the method of permuted blocks. It will be implemented using the REDCap randomization module. No one other than the statistician will have access to the block size(s). Eligible patients will be assigned to one of the following arms:

- Treatment with Relugolix combination therapy (Myfembree®) after surgical myomectomy.
- Treatment with standard of care (SoC) after surgical myomectomy.

Single vs. multi-center: Single center

2.2 Study objectives

2.2.1 The **primary outcome** will be a composite outcome including:

1. Fibroid recurrence when compared to the post-myomectomy baseline pelvic ultrasound (transvaginal and/or transabdominal), defined as a new fibroid identified on ultrasound with volume >1 cm^3 (Rossetti, Sizzi, & Soranna, 2001)
2. Symptom recurrence defined as either of the following:
   I. Heavy menses with PBAC score ≥ 120 for patients with pre-operative heavy menses (Higham JM, 1990)
   II. Pelvic pain during menses as measured on the Numerical Rating Scale NRS ≥ 4 at baseline (Korff M, 2000)
   III. Score ≥ 25 on symptom severity scale (Harding, Coyne , & Thompson, 2008)
3. Need for reintervention defined as need to undergo a procedure or surgery for treatment of uterine fibroids including repeat myomectomy, uterine fibroid embolization, radiofrequency ablation or hysterectomy.

2.2.2 The **secondary outcomes** will include:

1. Fibroid recurrence, compared to baseline pelvic ultrasound (transvaginal and/or transabdominal), defined as a new fibroid identified on ultrasound with volume >1 cm^3 (Rossetti, Sizzi, & Soranna, 2001)
2. Return of heavy menses defined as PBAC score ≥ 120 for patients with pre-operative heavy menses (Higham JM, 1990)
3. Need for reintervention defined as need to undergo a procedure or surgery for treatment of uterine fibroids including repeat myomectomy, uterine fibroid embolization, radiofrequency ablation.
4. Time to event (procedure or surgery) such as radio frequency ablation (RFA), myomectomy, hysterectomy, or uterine fibroid embolization (UFE).
5. Symptom recurrence according to the UF quality of life symptoms severity subscale and Bleeding & pelvic discomfort (BPD) scale.
6. Return of pelvic pain during menses as measured on NRS (Korff M, 2000)
7. Quality of life as measured by Patient Reported Outcome and Quality of life questionnaires (UFS-QOL, WPAI:SHP, Female Sexual Function Index)

2.3 Aims & Hypotheses

2.3a Specific Aim 1

To determine if there is a difference in fibroid recurrence (defined as a new fibroid identified on ultrasound with volume >1 cm^3), symptom recurrence (defined as either heavy menses with PBAC score ≥ 120, pelvic pain during menses measured on NRS ≥ 4 at baseline or score ≥ 25 on symptom severity scale) or need of reintervention between women receiving myomectomy plus Relugolix combination therapy compared to those receiving standard of care after myomectomy.

2.3a.1 Hypothesis 1

There will be decreased rate of fibroid recurrence (defined as a new fibroid identified on ultrasound with volume >1 cm^3), symptom recurrence (defined as either heavy menses with PBAC score ≥ 120, pelvic pain during menses measured on NRS ≥ 4 at baseline or score ≥ 25 on symptom severity scale) or need for reintervention in patients receiving myomectomy plus Relugolix combined therapy vs. standard of care after myomectomy.

2.3b Specific Aim 2

To determine if there is a difference in quality-of-life scores as measured by Patient Reported Outcome and Quality of life questionnaires (UFS-QOL, WPAI:SHP, Female Sexual Function Index,) in patients receiving myomectomy plus Relugolix combination therapy compared to those receiving standard of care after myomectomy.

2.3b.1 Hypothesis 2

There will be an improvement in quality-of-life scores as measured by Patient Reported Outcome and Quality of life questionnaires (UFS-QOL, WPAI:SHP, Female Sexual Function Index,) in patients receiving myomectomy plus Relugolix combined therapy vs. standard of care after myomectomy.

2.4 Data Management and Statistical Analysis

Data analysis and statistical support is obtained through the Biostatistics Laboratory (located within the Department of Public Health Sciences) at the University of Chicago. The primary endpoint of this study will be a composite endpoint (binary, Yes vs No) of the following three endpoints collected over a 24 month follow up period.
Endpoint 1 (Recurrence)
Endpoint 2 (Symptom)
Endpoint 3 (Re-intervention)
A Chi-square or Fisher's exact test will be used to test rate difference with a prior alpha of 0.05 and 80% power.

The assumption of endpoint 1 is based on (Rossetti, Sizzi, & Soranna, 2001). The study observed 23% recurrences in the abdominal group and 27% in the laparoscopic group over 40-month time. 84% of recurrence was seen within 24 months. Therefore, the recurrence rate among patients with laparoscopic or abdominal surgery over 24 month follow up time will be (23% +27%)/2*0.84=0.21. We assume a recurrence rate of 0.05 in the treatment group.
For endpoint 2, a symptom recurrence rate of 0.076 (Sangha R, 2016) (Manyonda I, 2020) in the control group and recurrence rate of 0.015 in the treatment group were assumed. For endpoint 3, a reintervention rate of 0.144 (Sangha R, 2016) in the control group and a recurrence rate of 0.03 in the treatment group were assumed for endpoint 3; endpoint 1 is independent of endpoints 2 and 3; and a correlation of 0.5 between the endpoints 2 and 3.

	Treatment Group rate	Control Group rate
Endpoint 1 (Recurrence)	0.05	0.21
Endpoint 2 (Symptom)	0.015	0.076
Endpoint 3 (Re-intervention)	0.03	0.144

Using the standard formula for the occurrence of either of 3 events: $Pr(A_1UA_2UA_3)=Pr(A_1)+Pr(A_2)+Pr(A_3)-Pr(A_1\cap A_2)-Pr(A_1\cap A_3)-Pr(A_2\cap A_3)+Pr(A_1\cap A_2\cap A_3)$,
Composite rate in control is -0.338;
Composite rate in treatment is 0.0825;
The sample size from PASS is 47 in each treatment arm.
Assume a 30% drop out rate: S*(1-0.3) = 94 -> S 94/0.7= 135 ~136. It is expected that the 136 patients will be accrued during the first year of the trial (10-12 per month) with two years of follow-up after the last patient is enrolled. The number needed to treat (NNT) will be calculated as the reciprocal of the difference in observed event rates along with a 95% confidence interval. As secondary endpoints we will analyze the three components of the primary endpoint (recurrence, symptoms, and re-intervention) separately using Chi-square or Fisher's exact test. Adverse event rates will be summarized by type, grade, and attribution and monitored periodically by the investigative team. Pain scores and quality of life (QOL) measures will be analyzed using mixed effects models for longitudinal data (Gibbons and Hedeker, 2000). While the sample size has been increased to allow for a 30% dropout rate, every effort will be made to retain subjects in the trial and to obtain outcomes data. Sensitivity analyses will be performed to examine the effects

of dropouts on the results.

2.4 Graphical schema of study

This will be a randomized, single center, open label interventional clinical trial of treatment with Relugolix combination therapy (Myfembree®) after surgical myomectomy vs. Treatment with standard of care **after** surgical myomectomy. The randomization scheme will be coordinated **by** the The Biostatistics Laboratory (located within the Department of Public Health Sciences) at the **U**niversity of Chicago.

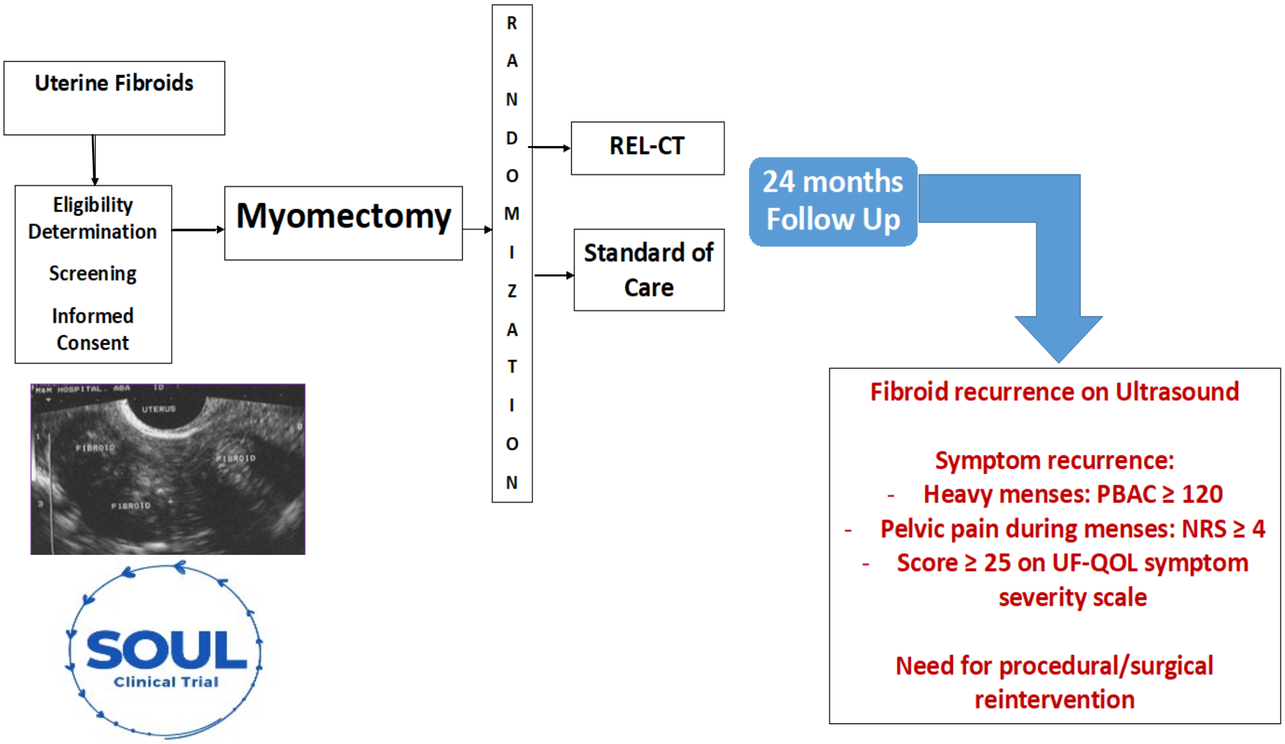

3.0 STUDY PROCEDURES

Patients who are scheduled for a myomectomy with their provider will be identified based on a review of their medical chart. Those patients will be approached to confirm that they meet the eligibility requirement for enrollment in the study. A total of 136 participants who are meet all the eligibility criteria will be consented and enrolled in the study. Eligibility criteria will be determined by a combination of medical chart review and a thorough discussion with potential participants. After myomectomy, patients will be randomized to receive Relugolix combination therapy, Myfembree (interventional study arm) or standard of care post myomectomy.

At 4 weeks post-surgery, participants will complete a baseline ultrasound. Participants in the medication arm will receive a once daily pill that contains Relugolix (a gonadotropin-releasing hormone antagonist), estradiol (an estrogen) and norethindrone acetate (a progestin). Myfembree will be dispensed after randomization at study visit 1. Pill counts and checks will be completed at each visit.

- A total of 10 visits are necessary including the screening visit.

All participants will be asked to fill out questionnaires at baseline and then every 3 months. The questionnaires will be filled electronically using uMotif ediary.

- Uterine Fibroid Symptom and Health Related Quality of Life (UFS-QoL) fibroid Questionnaire (Spies, Coyne, Guaou Guaou, 2002)
- Work Productivity and Activity Impairment Questionnaire: Specific Health Problem V2.0 (WPAI:SHP) (Reilly, Zbrozek, & Dukes, 1993)
- Female Sexual Function Index (FSFI) (Rosen, Brown, & Heiman, 2000)

All participants will be asked to fill a set of diaries at baseline and daily for the study duration

- Daily electronic diaries (eDiary) to measure compliance with study treatment
- Menstrual bleeding diary
- Record of use of feminine products for menstrual bleeding
- Pictorial bleeding assessment chart (PBAC) scores
- Pelvic pain during menses by the numeric rating scale (NRS)
- Use of pain medication to treat pain caused by uterine fibroids

Participants will have access to all questionnaires once they are enrolled in the study and they will be accessible anywhere through their phones, mobile or computer devices.

All participants will undergo pelvic ultrasounds at the following time periods:

- Week 4: post-myomectomy baseline ultrasound (transvaginal and/or transabdominal)
- Month 6: pelvic ultrasound (transvaginal and/or transabdominal)
- Month 12: pelvic ultrasound (transvaginal and/or transabdominal)
- Month 18: pelvic ultrasound (transvaginal and/or transabdominal)
- Month 24: pelvic ultrasound (transvaginal and/or transabdominal)

All participants will have blood work performed every 6 months or at clinically indicated intervals

- Cell Blood Count (CBC)
- Complete Metabolic Panel (CMP)
- Urine pregnancy test during each clinic/in-person visit.

**Procedures**	Screening (in person or virtual)	Study Visit 1 at 4 weeks (in person)	Study Visit 2 at 3 months (virtual)	Study Visit 3 at 6 months (in person)	Study Visit 4 at 9 months (virtual)	Study Visit 5 at 12 months (in person)	Study Visit 6 at 15 months (virtual)	Study Visit 7 at 18 months (in person)	Study Visit 8 at 21 months (virtual)	Study Visit 9 at 24 months (in person) (End of study)
Informed Consent	X									
Demographics	X									
Urine Pregnancy Test	X	X		X		X		X		X
Safety Labs (CBC+CMP)	X*			X**		X**		X**		X**
Medical History	X									X
Reproductive History	X									X
Physical Examination	X					X				X
WPAI:SHP Questionnaires	X		X	X	X	X	X	X	X	X
UFS-QOL Questionnaires	X		X	X	X	X	X	X	X	X
FSFI Questionnaire	X		X	X	X	X	X	X	X	X
E-diaries follow up	X	X	X	X	X	X	X	X	X	X
Randomization		X								
Myfembree drug dispensing		X	X	X	X	X	X	X	X	X
Pill Count/Compliance			X	X	X	X	X	X	X	X
Concomitant Medication Review	X	X		X		X		X		X
Adverse Event Review		X	X	X	X	X	X	X	X	X
Pelvic Ultrasound (transvaginal and/or transabdominal		X		X		X		X		X

* Or document work up done within six months of screening visit
**Or document safety labs done within the past 30 days.

4.0 STUDY DRUGS

Relugolix combination therapy (relugolix 40 mg, estradiol 1 mg, norethindrone 0.5 mg) once/day for 24 months post myomectomy. Relugolix combination therapy (relugolix with estradiol and norethindrone acetate, Myfembree®) received FDA approval to control heavy menstrual bleeding due to uterine fibroids in premenopausal women, for up to two years (Al-Hendy, Lukes, Poindexter, Venturella, & Villarroel, 2021).

According to available data Myfembree®, outperformed its placebo comparison groups with a total of 73% and 71% of study participants receiving relugolix combination therapy as compared to 19% and 15% of those in placebo groups, respectively, achieving the primary efficacy endpoint - volume of menstrual blood loss <80 ml and a ≥50% reduction in volume from baseline. Patients receiving Myfembree® versus placebo also showed significant improvements in six of seven key secondary endpoints - measures of menstrual blood loss (including amenorrhea), pain, distress from bleeding and pelvic discomfort, anemia, and uterine volume. These long-term, well- tolerated medical therapies are the first group of medications designed specifically for AUB-L and represent a changing paradigm in the fibroid treatment. Relugolix combination therapy, Myfembree® significantly reduced fibroid-related pain measured with the use of a daily electronic diary and a validated pain-outcome measure (Al-Hendy, Lukes, Poindexter, Venturella, & Villarroel, 2021)

5.0 STUDY INSTRUMENTS: Questionnaires (PDF attached to IRB application portal)

6.0 STUDY SUBJECTS

Target subjects are premenopausal females aged 18 years and older on the day of signing of a consent form. Subjects will consist of women of reproductive age with symptomatic fibroids who have decided, after counseling by a gynecologic surgeon, to proceed with myomectomy for treatment of symptomatic fibroids. Additionally, subjects will be patients who have decided not to conceive during the study treatment period which will be a total of 24 months following myomectomy.

6.1 Inclusion criteria:

Inclusion Criteria (all inclusion criteria must have been met prior to randomization unless otherwise specified):

1. Has voluntarily signed and dated the informed consent form prior to initiation of any screening or study-specific procedures
2. Premenopausal female aged 18 years and older on the day of signing of the informed consent form
3. Has a diagnosis of uterine fibroids that is confirmed by a pelvic ultrasound (transvaginal and/or transabdominal) performed during the screening period.
4. Has at least one or more of the following symptoms:
    a. Heavy menses defined as PBAC score ≥ 120
    b. Pelvic pain during menses measured on NRS ≥ 4 at baseline
    c. Moderately severe fibroid-related symptoms (a score ≥ 25 on the UF quality of life symptoms severity subscale)
5. Has a negative urine pregnancy test at the Screening, Baseline and interval clinic visits
6. Agrees to use two forms of non-hormonal contraception (dual contraception) consistently during the screening period and the randomized treatment period. These may include: Diaphragm, cervical cap, spermicides, male and female condoms, copper IUD and

sponge. Each one will be explained in detail for the participants. However, the patient is not required to use dual contraception if she:

a. Has a sexual partner(s) who was vasectomized at least 6 months prior to the screening period.
b. Had a bilateral tubal occlusion (including ligation and blockage methods such as Essure™), at least 4 months prior to the first screening visit (patients with Essure™ must have prior confirmation of tubal occlusion by hysterosalpingogram);
c. Is not sexually active with men; periodic sexual relationship(s) with men requires the use of dual non-hormonal contraception as noted above; or
d. Practices total abstinence from sexual intercourse as her preferred lifestyle; periodic abstinence is not acceptable.

7. Has an endometrial (aspiration) biopsy, if clinically indicated, performed during the screening period, with results showing no clinically significant endometrial pathology (hyperplasia, endometritis, or endometrial cancer).
8. If ≥ 40 years of age at the time of the Baseline Day 1 visit, has a normal mammogram (Breast Imaging Reporting and Data System category 1 to 3 or equivalent) during the screening period or within 12 months prior to the screening period.

6.2 Exclusion criteria:

1. Has transvaginal and/or transabdominal ultrasound during the screening period demonstrating pathology other than uterine fibroids that could be responsible for or contributing to the patient's heavy menstrual bleeding, such as uterine or cervical polyps >1.0 cm, large simple ovarian cyst >4.0 cm, endometrioma(s), or any other clinically significant gynecological disorder determined by the investigator to require further evaluation and/or treatment.

   Note: Saline or gel contrast is not routinely required. Use of such contrast is required only when the endometrium cannot be evaluated or when there are ambiguous and potentially exclusionary findings on the transvaginal and/or transabdominal ultrasound (e.g., suspected intrauterine masses, equivocal endometrial findings, etc.)

2. Has unexplained vaginal bleeding outside of the patient's regular menstrual cycle

3. Has undergone ultrasound-guided laparoscopic radiofrequency ablation, or any other surgical procedure for fibroids, uterine artery embolization, magnetic resonance-guided focused ultrasound for fibroids, as well as endometrial ablation for abnormal uterine bleeding within 6 months prior to the Screening visit
4. Has visually confirmed endometriosis diagnosis: detection of endometriotic lesions during laparoscopy or laparotomy (with or without pathological diagnosis) within the past 10 years.
5. Has a history of or currently has osteoporosis, or other metabolic bone disease, hyperparathyroidism, hyperprolactinemia, hyperthyroidism, anorexia nervosa, or low traumatic (from the standing position) or atraumatic fracture (toe, finger, skull, face, and ankle fractures are allowed). A history of successfully treated hyperparathyroidism, hyperprolactinemia, or hyperthyroidism is allowed if the patient's bone mineral density is within normal limits
6. Has a history of the use of bisphosphonates, calcitonin, calcitriol, ipriflavone, teriparatide, denosumab, or any medication other than calcium and vitamin D preparations to treat bone mineral density loss
7. Anticipated use of systemic glucocorticoids at an oral prednisone-equivalent dose of more than 5 mg every other day during the study. Note: topical, inhaled, intranasal, optic, ophthalmic, intraarticular, or intralesional subcutaneous are permitted without restriction

8. Gastrointestinal disorder affecting absorption or gastrointestinal motility
9. Has any additional contraindication to treatment with low-dose estradiol and norethindrone acetate, including:
    a. Current, known, suspected, or history of breast cancer
    b. Current, known, or suspected hormone -dependent neoplasia
    c. High risk of arterial, venous thrombotic disorder or thromboembolic disorder
        i. women over 35 years of age who smoke or women with uncontrolled hypertension
    d. Active thrombotic or thromboembolic disease or history of these conditions prior to the Baseline Day 1 visit or risk factors for such conditions. These conditions include:
        i. deep vein thrombosis
        ii. pulmonary embolism
        iii. vascular disease (e.g., cerebrovascular disease, coronary artery disease, peripheral vascular disease)
        iv. inherited or acquired hypercoagulopathies, known protein C, protein S, or antithrombin deficiency, or other known thrombophilia disorders, including Factor V Leiden thrombogenic valvular or thrombogenic rhythm diseases of the heart (for example, subacute bacterial endocarditis with valvular disease, or atrial fibrillation)
        v. uncontrolled hypertension
        vi. headaches with focal neurological symptoms or migraine headaches with aura if over 35 years of age
        vii. Women at increased risks for thrombotic or thromboembolic events

    e. Known anaphylactic reaction or angioedema or hypersensitivity to estradiol or norethindrone acetate
    f. Currently pregnant or lactating, or intends to become pregnant or to donate ova during the study period or within 1 month after the end of the study
10. Has jaundice or known current active liver disease from any cause, including hepatitis A (HAV IgM), hepatitis B (HBsAg), or hepatitis C (HCV Ab positive, confirmed by HCV RNA);
11. Has any of the following cervical pathology: high grade cervical neoplasia, atypical glandular cells, atypical endocervical cells, atypical squamous cells favoring high grade. Of note, patients with atypical squamous cells of undetermined significance and low-grade cervical neoplasia may be included in the study if high risk human papilloma virus testing is negative or if DNA testing for human papilloma virus 16 and 18 DNA testing is negative
12. Has any of the following clinical laboratory abnormalities indicating hepatic or gallbladder impairment:
    a. Alanine aminotransferase or aspartate aminotransferase > 2.0 times the upper limit of normal (ULN), or bilirubin (total bilirubin) > 1.5 x ULN on clinical laboratory testing at either the Screening 1 or Screening 2 visit (or > 2.0 x ULN if secondary to Gilbert syndrome or pattern consistent with Gilbert syndrome);
    b. Estimated glomerular filtration rate < 60 $mL/min/m^2$ using the Modification of Diet in Renal Disease method
13. Has clinically significant cardiovascular disease including:
    a. Prior history of myocardial infarction
    b. History of angina
    c. History of congestive heart failure
    d. History of clinically significant ventricular arrhythmias such as ventricular tachycardia, ventricular fibrillation, or torsade de pointes, or Mobitz II second

degree or third-degree heart block without a permanent pacemaker in place or untreated supraventricular tachycardia (heart rate ≥ 120 beats per minute)

e. QT interval by the Fridericia correction formula (QTcF) of > 470 msec
f. Hypotension, as indicated by systolic blood pressure < 84 millimeters of mercury (mmHg) on 2 repeat measures at least 15 minutes apart or treated ongoing symptomatic orthostatic hypotension with > 20 mmHg decrease in systolic blood pressure one minute or more after assuming an upright position.
g. Uncontrolled hypertension, as indicated by systolic blood pressure > 160 mmHg on 2 repeat measures at least 15 minutes apart or diastolic blood pressure > 100 mmHg at any screening visit or the Baseline Day 1 visit.
h. Bradycardia as indicated by a heart rate of < 45 beats per minute on the screening electrocardiogram.

14. Has been a participant in an investigational drug or device study within the 1 month prior to Screening visit.
15. Has a history of clinically significant condition(s) including, but not limited to:
    a. Untreated thyroid dysfunction or palpable thyroid abnormality (patients with adequately treated hypothyroidism who are stable on medication are not excluded).
    b. History of malignancy within the past 5 years or ongoing malignancy other than curatively treated nonmelanoma skin cancer or surgically cured Stage 0 in situ melanoma
16. Any current psychiatric disorder that would, in the opinion of the investigator or medical monitor, impair the ability of the patient to participate in the study or would impair interpretation of their data. Patients with major depression, post-traumatic stress disorder, bipolar disorder, schizophrenia, or other psychotic disorders, based on Diagnostic and Statistical Manual of Mental Disorders-5 criteria who have been unstable based on the investigator's or mental health professional's judgement or whose psychiatric drug regimen has changed during the 3 months prior to Screening or is expected to change during the study should not be enrolled. Has a contraindication or history of sensitivity to any of the study treatments or components thereof; or has a history of drug or other allergy that, in the opinion of the investigator or medical monitor, contraindicates study participation
17. Has a prior (within 1 year of Screening 1 visit) or current history of drug or alcohol abuse disorder according to Diagnostic and Statistical Manual of Mental Disorders V (all patients must be questioned about their drug and alcohol use, and this should be documented in the electronic case report form)
18. Has participated in a previous clinical study that included the use of Relugolix or has received this treatment within 3 months of the study.
19. Is an immediate family member, study site employee, or is in a dependent relationship with a study site employee who is involved in the conduct of this study (e.g., spouse, parent, child, or sibling)
20. Is inappropriate for participation in this study for other reasons, as determined by the investigator, sub-investigator, or medical monitor.

7.0 RECRUITMENT

Gynecology surgeons who perform myomectomies and have agreed to participate in study recruitment will be asked for their permission to consider their patients for the study.

A member of the research team will search the University of Chicago operating room schedules

of the **surgeons** to identify potential study participants scheduled to undergo a myomectomy. Data from the medical record system will be used to evaluate inclusion criteria. **Patients** presenting **to the clinic** for a consultation for fibroid symptoms or fibroid management (after clinical confirmation of their eligibility for admission into the study based on inclusion & exclusion criteria) will also be considered.

Once a potential participant is identified, they will be approached to participate **by** a member of the research team. Contact is established via mychart, email, phone number, in person at the clinic or pre-op. Eligibility criteria will be confirmed by reviewing medical history and thorough discussion with potential subject. If eligibility is confirmed, the potential subject will be offered participation in the study. At this **time,** study details **will** be explained. Risks and benefits will be thoroughly discussed, and consents given to the participant. Details of the consenting process will be discussed later in section 8.0.

8.0 INFORMED CONSENT PROCESS

After a member of the research staff determines eligibility by reviewing medical history and thorough discussion with study participants, the informed consent process will be completed in three possible ways as listed/explained below by the PI or research staff (Co-investigator or other delegated study staff):

Option 1: (in person consent)

- Subject will be offered an in person visit with a member of the research staff.
- During this visit, the informed consent and HIPAA form will be explained, and all questions answered
- The informed consent and HIPAA form will be signed by the patient and a copy given to them for their personal records
- The original 'wet-ink' signed consent and HIPAA form will be stored in a locked cabinet in the research coordinator's office.
- All inclusion and exclusion criteria must be met to be enrolled in the study.
- All preoperative data, and screening visit events will be obtained as previously outlined in the study procedures section.

Option 2: (phone discussion with paper consent form)

- Subject will be offered a phone discussion. Prior to this phone discussion a copy of the consent form will be mailed to the subject. Receipt of consent will be verified before the phone consenting process begins.
- During this conversation the informed consent and HIPAA form will be explained, and all questions answered
- Two copies of the consent and a stamped, addressed envelope will be mailed to the patient to sign and return the consent form.
- All inclusion and exclusion criteria must be met to be enrolled in the study. Then the subject will receive study materials by mail or email

Option 3: (phone discussion with electronic consent form)

- Subject will be offered a phone discussion. An email with a link / instruction for accessing REDCap will be sent to the subject. They will be instructed to access the electronic form to review it during the consenting process.
- During this conversation the informed consent and HIPAA form will be explained, and all

questions answered
- The patient will sign the electronic consent form accessed through REDCap.
- Once the consent form is completed electronically the form will be printed and kept in a locked filling cabinet for our records.
- All inclusion and exclusion criteria must be met to be enrolled in the study. A copy of the signed consent form and the study medication and/or materials will be mailed to the subject.

For all options, participants will have access to the consent form prior to and during the consenting process. In addition, when participation is offered to the patient, the informed consent and HIPAA form will be explained in detail along with the study design, procedures, inclusion/exclusion criteria, risks and benefits, and study requirement. It will be emphasized that participation is completely voluntary and that she may revoke her participation at any time. There will be no further documentation of the informed consent process.

9.0 CONFIDENTIALITY OF STUDY DATA

To ensure confidentiality of medical information, each patient will be assigned a unique identifier in the database that can be linked to the medical record number. The database will be password-protected, encrypted and stored on a secure server accessible only from computers in the OBGYN department. Subject demographics and date will be entered into REDCap (system 4283). REDCap is a mature and secure web application for building and managing online surveys and databases. It allows data to be exported to Excel or R or SPSS.

REDCap has the capacity for allowing patients to securely link to consent forms. The electronic consent form will be accessed via an email link. REDCap is HIPAA compliant, and data is encrypted during transmission, allowing for data collection out-of-network, off-campus, remotely, etc. Patients are able to sign the consent for with the use of a mouse or a touch screen.

UMotif is BSI certified and operates a quality management system that is ISO9001-2015 compliant. It is used for patient centric software as a service platform to capture electronically clinical outcome assessment data in a healthcare setting including healthcare industry.

10.0 PRIVACY PROTECTIONS

The Principal Investigator and study staff will assure that the subject’s privacy will be strictly maintained and that their identities are protected from unauthorized parties. This will be accomplished by securing all study documents and subject information. These files will be accessible to study staff only and maintained in a secure study office. The study staff will assign a code (numbers and/or letters) to the subject for data analysis. Documents that contain identifiers will be kept in a locked research office and/or stored within computers with password protection and encryption. We will safeguard patients’ expectation that the information they offer will be held in confidence. We will protect each participant’s information as prescribed by the University and Hospital policy and relevant Federal law.

11.0 POTENTIAL RISKS

According to available data, this study has minimal risks. Myomectomy related risks will be discussed in detail by your surgeon. Participants in the standard of care (SoC) arm of the study have no additional risks because of this study. They will need to spend some time completing the diaries and questionnaires as well as undergo periodic blood work and pelvic ultrasound. All of which are unlikely to be of major clinical risk but may cause discomfort (ultrasound) or bruising (blood work).

Blood Draw risks: Blood draw is routine in most laboratory investigations. Risks of blood draw include pain, bruising at the site of blood draw, redness and swelling of the vein and infection, and a rare risk of fainting. These risks will be minimized by having a certified phlebotomist or experienced RN draw the lab orders.

Pelvic ultrasound risks: abdominal, vaginal or pelvic discomfort

Risks related to filling out the questionnaires may make the patient uncomfortable or feel some anxiety. There is a minimal risk that information about the patient may become known to people outside of this study.

Myfembree related risks:

In a study comparing patients on Myfembree to those taking placebo, patients receiving Myfembree were at a slightly increased risk of abnormal uterine bleeding (6.3% vs. 1.2%), hot flushes or night sweats (10.6% vs. 6.6%), decreased libido (3.1% vs. 0.4%) hair thinning (3.5% vs. 0.8%), new or worsening hypertension (7.0% vs. 0.8%).

As with all estrogen and progestin combination products, Myfembree increases the risk of deep vein thrombosis (DVT), pulmonary embolism (PE), stroke, myocardial infarction. These risks are less likely in women with low baseline risk of these events. Therefore, rigorous screening is performed before starting this medication.

PRECAUTIONS & WARNINGS WITH MYFEMBREE INCLUDE:
OF NOTE Exclusion and Inclusion Criteria are designed to minimize the risks of Myfembree

Thromboembolic Disorders: Discontinue immediately if an arterial or venous thrombotic, cardiovascular, or cerebrovascular event occurs or is suspected. Discontinue at least 4 to 6 weeks before surgery associated with an increased risk of thromboembolism, or during periods of prolonged immobilization, if feasible. Discontinue immediately if there is sudden unexplained partial or complete loss of vision, proptosis, diplopia, papilledema, or retinal vascular lesions and evaluate for retinal vein thrombosis as these have been reported with estrogens and progestins.

Bone Loss: Myfembree may cause a decrease in bone mineral density (BMD) in some patients, which may be greater with increasing duration of use and may not be completely reversible after stopping treatment. Consider the benefits and risks in patients with a history of low trauma fracture or risk factors for osteoporosis or bone loss, including medications that may decrease BMD. Assessment of BMD by dual-energy X-ray absorptiometry (DXA) is recommended at baseline and periodically thereafter. Consider discontinuing Myfembree if the risk of bone loss exceeds the potential benefit.

Hormone-Sensitive Malignancies: Discontinue Myfembree if a hormone-sensitive malignancy is diagnosed. Surveillance measures in accordance with standard of care, such as breast examinations and mammography are recommended. Use of estrogen alone or estrogen plus progestin has resulted in abnormal mammograms requiring further evaluation.

Depression, Mood Disorders, and Suicidal Ideation: Promptly evaluate patients with mood changes and depressive symptoms including shortly after initiating treatment, to determine whether the risks of continued therapy outweigh the benefits. Patients with new or worsening depression, anxiety, or other mood changes should be referred to a mental health professional, as appropriate. Advise patients to seek immediate medical attention for suicidal ideation and behavior and reevaluate the benefits and risks of continuing Myfembree. We will follow HIPAA guidelines and State laws for physicians' "duty to warn" if there is a serious and imminent threat of a patient physically harming self or others.

Hepatic Impairment and Transaminase Elevations: Steroid hormones may be poorly metabolized in these patients. Instruct women to promptly seek medical attention for symptoms or signs that may reflect liver injury, such as jaundice or right upper abdominal pain. Acute liver test abnormalities may necessitate the discontinuation of Myfembree use until the liver tests return

to normal and Myfembree causation has been excluded.

Gallbladder Disease or History of Cholestatic Jaundice: Discontinue Myfembree if signs or symptoms of gallbladder disease or jaundice occur. For women with a history of cholestatic jaundice associated with past estrogen use or with pregnancy, assess the risk-benefit of continuing therapy. Studies among estrogen users suggest a small increased relative risk of developing gallbladder disease.

Elevated Blood Pressure: For women with well-controlled hypertension, monitor blood pressure and stop Myfembree if blood pressure rises significantly.

Change in Menstrual Bleeding Pattern and Reduced Ability to Recognize Pregnancy: Advise women to use non-hormonal contraception during treatment and for one week after discontinuing Myfembree. Avoid concomitant use of hormonal contraceptives. Myfembree may delay the ability to recognize pregnancy because it alters menstrual bleeding. Perform testing if pregnancy is suspected and discontinue Myfembree if pregnancy is confirmed.

Risk of Early Pregnancy Loss: Myfembree can cause early pregnancy loss. Exclude pregnancy before initiating and advise women to use effective non-hormonal contraception.

Uterine Fibroid Prolapse or Expulsion: Advise women with known or suspected submucosal uterine fibroids about the possibility of uterine fibroid prolapse or expulsion and instruct them to contact their physician if severe bleeding and/or cramping occurs.

Alopecia: Alopecia, hair loss, and hair thinning were reported in phase 3 trials with Myfembree. Consider discontinuing Myfembree if hair loss becomes a concern. Whether the hair loss is reversible is unknown.

Effects on Carbohydrate and Lipid Metabolism: More frequent monitoring in Myfembree-treated women with prediabetes and diabetes may be necessary. Myfembree may decrease glucose tolerance and result in increased blood glucose concentrations. Monitor lipid levels and consider discontinuing if hypercholesterolemia or hypertriglyceridemia worsens. In women with pre-existing hypertriglyceridemia, estrogen therapy may be associated with elevations in triglycerides levels leading to pancreatitis. Use of Myfembree is associated with increases in total cholesterol and LDL-C.

Effect on Other Laboratory Results: Patients with hypothyroidism and hypoadrenalism may require higher doses of thyroid hormone or cortisol replacement therapy. Use of estrogen and progestin combinations may raise serum concentrations of binding proteins (e.g., thyroid-binding globulin, corticosteroid-binding globulin), which may reduce free thyroid or corticosteroid hormone levels. Use of estrogen and progestin may also affect the levels of sex hormone-binding globulin, and coagulation factors.

Hypersensitivity Reactions: Immediately discontinue Myfembree if a hypersensitivity reaction occurs.

ADVERSE REACTIONS:

Most common adverse reactions for Myfembree (incidence ≥3% and greater than placebo) were hot flush/hyperhidrosis/night sweats, abnormal uterine bleeding, alopecia, and decreased libido. These are not all the possible side effects of Myfembree.

DRUG INTERACTIONS:

P-gp Inhibitors: Avoid use of Myfembree with oral P-gp inhibitors. If use is unavoidable, take Myfembree first, separate dosing by at least 6 hours, and monitor patients for adverse reactions. **Combined P-gp and Strong CYP3A Inducers:** Avoid use of Myfembree with combined P-gp and strong CYP3A inducers.

LACTATION: Advise women not to breastfeed while taking Myfembree.

12.0 ADVERSE EVENTS DEFINITION

An AE is any untoward medical occurrence temporally associated with the use of a medicinal product. An AE can be any unfavorable and unintended sign (for example, an abnormal laboratory finding), symptom, or disease. Investigators, or a designee, will document AEs. All AEs must be recorded on an AE Form of Redcap. Adverse events will be coded using the MedDRA AEs dictionary. All AEs will be reported in the participant's source documents regardless of relationship to the study product. All AEs will be followed during the trial period. The Investigator will use his discretion in ordering additional tests as necessary to monitor the resolution of such events. Event outcome at resolution or at time of last follow up will be recorded as event resolved, resolved with sequelae, ongoing at discontinuation, or death. Any events ongoing after the participant's last visit will be followed by attempting to contact the participant at a minimum for up to 28 days after the visit to determine status. Beyond that time frame, Investigators should continue to follow up events of concern outside of the trial per their standard of care. The trial database will be finalized in these cases with events noted as ongoing. The Investigator should consider AEs both as they relate to Relugolix combination therapy, Myfembree, and as they relate to the procedures involved in the trial. The Investigator is responsible for determining initial relationship and severity of AEs.

12.1 Definition of Serious Adverse Events (SAE)

A serious adverse event (SAE) is defined by federal regulation as any AE occurring at any dose that results in any of the following outcomes: death, life-threatening AE, requires hospitalization or prolongation of existing hospitalization, a persistent or significant disability/incapacity, or a congenital anomaly/birth defect.
Important medical events that may not result in death, be life threatening, or require hospitalization may be considered an SAE when, based upon appropriate medical judgment, they may jeopardize the participant and may require medical or surgical intervention to prevent one of the outcomes listed in this definition. Examples of such medical events include allergic bronchospasm requiring intensive treatment in an emergency room or at home, blood dyscrasias or convulsions that do not result in participant hospitalization, or the development of drug dependency or drug abuse.

12.2. Classification of an Adverse event

12.2.1. Severity of Event

The severity of all AEs will be assessed by the Investigator and classified as mild, moderate, or severe. Severity will be graded according to the following definitions:

• Mild: The participant experiences awareness of symptoms but these are easily tolerated or managed without specific treatment.

• Moderate: The participant experiences discomfort enough to cause interference with usual activity, and/or the condition requires specific treatment.

• Severe: The participant is incapacitated with inability to work or do usual activity, and/or the event requires significant treatment measures.

12.3. Relationship to Study Intervention

All AEs must have their relationship to study intervention assessed by the clinician who examines and evaluates the participant based on temporal relationship and his clinical judgment. The degree of certainty about causality will be graded using the categories below. In a clinical trial, the study product must always be suspect.

• Related – There is evidence to suggest a causal relationship, and the influence of other factors is unlikely. The clinical event, including an abnormal laboratory test result, occurs within a reasonable time after administration of the study intervention, is unlikely to be attributed to concurrent disease or other drugs or chemicals, and follows a clinically reasonable response on withdrawal (rechallenge). Rechallenge information is not required to fulfill this definition.

• Not Related – A clinical event, including an abnormal laboratory test result, whose temporal relationship to study intervention administration makes a causal relationship improbable (e.g., the event did not occur within a reasonable time after administration of the study intervention) and in which other drugs or chemicals or underlying disease provides plausible explanations (e.g., the participant's clinical condition, other concomitant treatments). Or the AE is completely independent of study intervention administration, and/or evidence exists that the event is related to another etiology. There must be an alternative, definitive etiology documented by the clinician.

12.4. Time Period and Frequency for Event Assessment and Follow-up

The occurrence of an AE or SAE may come to the attention of study personnel during study visits and interviews of a study participant presenting for medical care, or upon review by a study monitor. All AEs including local and systemic reactions not meeting the criteria for SAEs will be captured in an AE Case Report Form in REDCap. Information to be collected includes event description, date of onset, clinician's assessment of severity, relationship to study product (assessed only by those with the training and authority to make a diagnosis), and date of resolution or stabilization of the event. All AEs occurring while on study must be documented appropriately regardless of relationship. Any medical condition that is present at the time that the participant is screened will be considered as baseline medical history and not reported as an AE. However, if the study participant's condition deteriorates at any time during the study, it will be recorded as an AE. Changes in the severity of an AE will be documented to allow an assessment of the duration of the event at each level of severity to be performed. Adverse events characterized as intermittent require documentation of onset and duration of each episode.

The Investigator will record all reportable events with start dates occurring any time after informed consent is obtained until the final study visit. At each study visit, the investigator will inquire about the occurrence of AEs/SAEs since the last visit. Event outcome at resolution or at time of last follow up will be recorded as "event resolved", "resolved with sequelae", "ongoing at

discontinuation", or "death".

12.5. Adverse Event Reporting

Disease related events expected in this population may be reported by the Investigator if they differ in nature or frequency from what is expected, or if they appear to have a causal relationship to the medication. All AEs must be reported in the participant's source documents and recorded on an AE Case Report Form regardless of whether or not they are related to the medication.

12.6. Serious Adverse Event Reporting

In the event of any SAE reported or observed during the trial, whether or not attributable to the trial medication, the Investigator, or designee, shall report the event to the medical monitor within 5 days being made aware of the event. An initial report should be made with the understanding that it may be lacking in detail.

A SAE Form must be completed for all SAEs and submitted within 5 days of the Investigator's knowledge of the event and to the Institutional Review Board according to their reporting requirements. When new significant information is obtained as well as when the outcome of an event is known, the Investigator must provide this information to the Sponsor as soon as it becomes available. Depending on the nature of the SAE, copies of the participant's medical records as well as results of any relevant laboratory tests performed maybe required to be submitted. If the participant was hospitalized, a copy of the discharge summary should be forwarded as soon as it becomes available. In certain cases, a letter from the Investigator that summarizes the events related to the case may be required.

12.7. Reporting Events to Participants

Any important risk that will change the risk-to-benefit ratio will be conveyed to study participants through updated consent forms. Study results will be shared with participants.

13.0 DATA AND SAFETY MONITORING

Study documents and subject information will be collected in files designed specifically for the study. These files will be accessible to study staff only and maintained in a locked and secure research study office. Electronic data will be stored in a mature and secure web application for building and managing online surveys and databases. Study personnel are trained and follow the data management guidelines of Good Clinical Practice to ensure confidentiality of medical information, each patient will be assigned a unique identifier in the database that could be linked to the medical record number. The database will be password-protected, encrypted and stored on a secure server accessible only from computers in the OBGYN Division. For data analysis, all stored data will be exported to Excel and well-established statistical analysis software such as R and SPSS. In order to comply with Good Clinical Practice (GCP) requirements, the investigators must maintain the master participant log that identifies all participants entered into the study for a period of two years after the study ends so that the participants can be identified by audit. The PI must maintain adequate records pertaining to participants' files and other source data for a minimum of 5 years after completion of the study.

14.0 POTENTIAL BENEFITS

Patients in the intervention arm may experience delay recurrence of fibroid symptoms, prolong improved quality of life and delay need for re-intervention after uterine sparing surgery. There are otherwise no additional potential benefits to participants of this study.

15.0 ALTERNATIVES

As an alternative to being in this study, you may choose not to participate. Your physician will continue to follow up with you and update you on any need for medical or surgical intervention as needed. Some options may include birth control pills, non-contraceptive hormones, medications like Myfembree and removing your uterus.

16.0 RESEARCH AT EXTERNAL SITES

Not Applicable

17.0 UNIVERSITYOF CHICAGO AS LEAD INSTITUTION

This is a single center study, with recruitment from University of Chicago Medical Center and a few satellite sites: Locations: The study patients will be recruited from outpatient settings of University of Chicago Medical Center:

University of Chicago DCAM - 5758 S. Maryland Ave Chicago, IL 60637

University of Chicago South Loop - 1101 S. Canal St. Suites 201 & 202 Chicago, IL 60607

University of Chicago Orland Park - 14290 S. La Grange Rd. Orland Park, IL 60462

University of Chicago Schererville: 222 Indianapolis Blvd. Schererville, IN 46375

University of Chicago River East: 355 E Grand Ave, Chicago, IL 60611

University of Chicago Flossmoor: 19550 Governors Hwy. Flossmoor, IL 60422

Proposed Timeline

Overall timelines: 36 months (3 years overall)
Date of initiation of study: 10/15/2022
Date of study enrollment completion: 10/15/2023
Follow up of last patient: 10/15/2025
Date of completion of analysis and submission of publication: 04/15/2026

Bibliography

Al-Hendy, A., Lukes, A. S., Poindexter, A. N., Venturella, R., & Villarroel, C. (2021). Treatment of Uterine FIbroid Symptoms with Relugolix Combination Therapy. *New E gland Journal of Medicine*, 384(7):630.

Candiani , G. B., Fedele, L., & Parazzini, F. (1991). Risk of recurrence after myomectomy. *Br J Obstet Gynaecol.* , 98 (4); 385 - 389.

Davis, M. R., Soliman, A. M., & Castelli-Haley, J. (2018). Reintervention Rates After Myomectomy, Endometrial Ablation, and Uterine Artery Embolization for Patients with Uterine Fibroids. *Journal of Women's Health*, 27:10, 1204-1214.

Donnez, J., Donnez, O., & Dolmans, M. (2014). With the advent of selective progesterone receptor modulators, what is the place of myoma surgery in current practice? *Fertil Steril*, 102 (3), 640-648.

Fauconnier, A., Chapron, C., & Babaki-Fard, K. (2000). Recurrence of leiomyomata after myomectomy. *Hum Reprod Update*, 6(6); 595-602.

Fedele, L., Vercellini, P., & Bianchi, S. (1990). Treatment with GnRH agonists before myomectomy and the risk of short-term myoma recurrence. *Br J Obstet Gynaecol.* , 97(5): 393-396.

Harding, G., Coyne , K. S., & Thompson, C. L. (2008). The responsiveness of the uterine fibroid symptom and health-related Quality of life questionnaire (UFS-QOL). *Health Qual Life Outcomes*, 6:99.

Higham JM, O. P. (1990). Assessment of menstrual blood loss using a pictorial chart. *Br J Obstet Gynaecol .*, 97: 734–739.

Korff M, J. M. (2000). Assessing global pain severity by self-report in clinical and health service sresearch. *Spine*, 25:3140-3151.

Lee, C.-L., & Wang, C.-J. (2009). Laparoscopic Myomectomy. *Taiwan J Obstet Gynecol*, 48(4): 335-41.

Liu, X., Tang, J., & Luo, Y. (2020). *Comparison of high-intensity focused ultrasound ablation and secondary myomectomy for recurrent symptomatic uterine fibroids following myomectomy: a retrospective study.* Retrieved from https://doi.org/10.1111/1471-0528.16262. 127:1422-1428

Manyonda I, B. A. (2020). FEMME Trial. *New England Journal of Medicine*, 383: 440 - 451.

Marjoribanks, J., Lethaby, A., & Farquhar, C. (2016). Surgery versus medical therapy for heavy menstrual bleeding. *Cochrane Database of SYstematic Reviews*, Issue 1.Art. No.:CD003855 DOI:10.1002/14651858.CD003855.

Munro MG, C. H. (2010). FIGO classification system (PALM-COEIN) for causes of abnormal uterine bleeding in nongravid women of reproductive age. *International Journal of Gynecology & Obstetrics*, 113: 3-13.

Nezhat, F. R., Roemisch, M., & Nezhat, C. H. (1998). Recurrence rate after laparoscopic myomectomy. *J Am Assoc Gynecol Laparosc*, 5: 237-240.

Rossetti, A., Sizzi, O., & Soranna, L. (2001). Long-term results of laparoscopic myomectomy: recurrence rate in comparison with abdominal myomectomy. *Hum Reprod*, 16: 770-774.

Sangha R, K. V. (2016). Recurrence after robotic myomectomy: is it associated with use of GnRH agonist? *J Robotic Surg*, 10, 245 - 249.

Stewart, E. A. (2001). Uterine Fibroids. *Lancet*, 357:293-8.

Sukur, Y. E., Kankaya, D., & Ates, C. (2015). Clinical and histopathologic predictors of reoperation due to recurrence of leiomyoma after laparoscopic myomectomy. *International Journal of Gynecology & Obstetrics*, 129; 75-78.
